# The Chicago Academy of Medicine

**Published:** 1898-10

**Authors:** 


### CORRESPONDENCE.

### THE CHICAGO ACADEMY OF MEDICINE.

CHICAGO, September 4, 1898.

MR. EDITOR:—The recently issued catalogue of the Chicago College of "Osteopathy" contains the assertion that the "orificialist surgeon," E. H. Pratt, one of its faculty, is a member of the Chicago Academy of Medicine. He has never been a member, nor is he eligible. He is probably a member of the Chicago Academy of Homeopathic Physicians and Surgeons, an organization with totally different objects and purposes.

Kindly publish this in justice to the Chicago Academy of Medicine. JAS. G. KIERNAN, *Secretary.*

---

MINERAL SPRINGS, ARK., August 20, 1898.

*Editor Atlanta Medical and Surgical Journal:*

SIR:—In reply as to the color of new-born negroes, I would state, in the experience of twenty years I have never seen one that could not readily be distinguished from a white infant. Generally they are very light at birth, but not of the pink color of the whites, and they *always* have a dark area around the anus. The lightest are, of course, of mixed blood, but I have seen real black ones at birth. Yours truly, T. J. DRAPER, M.D.

---

GIFFORD, S. C., September 20, 1898.

*Editor Atlanta Medical and Surgical Journal:*

I report this case, as I have never read nor heard of such in medical literature: On September 11, Lizzie McT., colored, gave birth to triplets; the first, a girl, was born about 11 o'clock A.M., the other two, one a boy the other a girl, not until eleven hours afterwards; the two last children were born within fifteen minutes of each other, and in about thirty minutes the placenta was delivered; the three placentas were attached to each other. I was called in to see the case ten hours after the first child was born, there only being a midwife with the patient when I was called. The history of the case was, that the patient had a fall from a fence about four feet high, and complained of pain the entire time after her fall, for about three weeks until her labor took place. The second two children were delivered by forceps. The first child lived for about thirteen hours, the other two died in about thirty minutes. The woman gave the history of being pregnant about seven months previous to her fall. There was no more hemorrhage than usual. The patient has never complained of any pain since her labor, and she is walking about apparently as well as ever. The os of the uterus had to be dilated with a Goodell's dilator before the second two children were born, as the internal os was firmly contracted, and they were delivered in the course of fifteen or twenty minutes after the os was dilated.

1. What could have caused the internal os to contract after the first child was born and prevent the delivery of the other two?

2. What caused the labor to be delayed so long after the fall?

Very truly yours, J. S. GREENLEAF, M.D.

